# The Stability of a Two-Axis Gimbal System for the Camera

**DOI:** 10.1155/2021/9958848

**Published:** 2021-05-06

**Authors:** Nguyen Cong Danh

**Affiliations:** The Independent Researcher, District 2, Ho Chi Minh City (HCMC), Vietnam

## Abstract

Gimbal or an inertial stabilization platform (ISP) is used to stabilize the line of sight of an object or device that is tracking another object (LOS) with stationary or moving targets or targets moving forward. It can achieve precision when there is isolation between the tracker and the gimbal base. Studying the 2-axis tilt angle to create gimbal stability, especially in a camera, is a compelling subject for the automation field, as it is controlled by modern controllers. This paper presents a two-axis gimbal loop in which the LOS rate is stable, and I proceed to examine the stability of the system to get a better overview of the system properties. Through examining the stability of the system, I can choose from modern control methods to control them. The stability of the system used from the two analysis methods I present below gives me a visual view from the results achieved. The simulation is performed in MATLAB.

## 1. Introduction

The gimbal is usually an observational device used in a variety of industries such as education, security, and communications. The key observer for the gimbal is usually a sensor such as a camera, but it can be anything such as an observatory's telescope and parabolic antenna. This requires the control system to aim most clearly for the target and the system to be modernized, which means more flexibility in the angle-of-view adjustment. A gimbal consists of revolutions at angles that are perpendicular to each other. A gimbal can stabilize an object or multiple built-in objects on it, or it can accommodate subloads for single or multiple spindles. A two-axis gimbal can stabilize an object or multiple subobjects built into it. The gimbal is sometimes considered a platform for inertial stability (ISP). Thus, the 2-axis system can be seen as a basic standard for building other complex gimbal systems. Recently, there are many studies that have been made on the model of automatic controllers in the field of automation and it is controlled by single gimbal and the multiaxis gimbal. For a multiaxis gimbal, there must be research work to develop software that specializes in making graphics and calculating complex structures for related models.

In three dimensions, the system needs to be designed with multiple gimbal axes to suit the purpose of use: I adjust the angle of view or the direction of the target to have the most accuracy even though the device moves at high speed in the air and it is not moving in a specific direction, based on the article of Obiora and Achumba [1]. Dynamic modeling of the gimbal in the universe or in the atmosphere of the planets is performed using independent joint control. The technique mentioned above will be controlled remotely via a computer or remote controller built into the flying device in the air or from another place on the ground. The independent joint control is independent and does not affect other joints or other actuators, to reduce the ability of the operator to make adjustments to other joints or actuators. However, this requires the designer to have a high level of expertise and knowledge in the field of independent joint control. In the future, there will be many studies for the multiaxis gimbal system that can be applied to flying devices to improve the quality of the system compared to the two-axis gimbal, as a new suggestion from the article Khayatian and Arefi [2]. I have drawn the conclusion of the article of Sasaki et al. [3]; the studies for scheduled controllers which are designed for multiaxis (more than 3 axes) are still limited because of the complexity of the problem, including the algorithm. My application of gimbal's achievements in automation and industry is a promising industry where I can apply different algorithms to different production equipment or tools, based on the article of Li et al. [4] . In the future, I can apply algorithms such as neural networks or adaptive algorithms to the system in the paper of Li et al. [5].

As for the development of this model in the paper of Caponetto and Xibilia [6], I can apply the multiaxis gimbal to the system. I can also integrate a Time Delay in addition to the adaptive controller or optimal controller, etc. for the models of the system in the paper written by Cui et al. [7]. Techniques to control the operational stability of the system in the work of Ahi and Nobakhti [8] as well as techniques to control the stability of the integrated controllers in the system need to be studied and implemented as a priority case. With methods of anti-interference involving harmonics, I can apply electrical engineering algorithms to suppress the noise for a period of time, based on the article of Huang et al. [9]. I can apply synthetic techniques from the paper of Fang and Ren [10] to test high precision through experiments about the target points of the system. Another proposal for the paper of Ding et al. [11] is the adaptive control using neural networks for the abovementioned model. Through Abdo et al.'s model [12], I propose the model using the PI control design method, and I will compare the advantages and disadvantages between PID controller and PI controller. Through Zhan et al.'s model [13], I can conduct a survey for this system with multiaxis gimbals (more than 2 axes). To develop the model in the paper of Majumder et al. [14], another proposal is the use of built-in techniques to control speed in harsh environments such as snow or fog and cloudy weather to achieve the exact goal.

To create a new topic for the model in the paper of Abdo et al. [15], I propose to use the PI controller. Using the technique, as a new proposal of the paper of Li et al. [16], I can conduct surveys for a 3-axis gimbal system. I can also suggest a fresh one from the model by Rajesh and Ananda [17], and I use a PI controller or a PID controller designed using a neural network to control the camera. With Lee and Yoo's model [18], I was able to refine it into 3-axis gimbal. Similar to the model of Lee and Yoo (2008), I can refine Lee's model [19] into a 3-axis gimbal seeker system. With the model in the paper of Kim et al. [20], the improvement is made with Lead-PID and H∞ robust controllers. I can incorporate a PID controller into the system and a PI controller into the system to enhance the diversification of uses, based on the model in the paper of Guo et al. [21]. To develop the model of Zheng and Han [22], I is used the techniques mentioned above to probe the angular velocity and attitude measurements for a drone. In Chen et al.'s work [23], they propose this model in the presence of a gyroscope and examine its performance. In the article by Li et al. [24], another improvement to the model is the use of a PID controller for system. I can use a PI controller for the system to investigate the behavior of Abdo et al.'s model [25]. In the model written by K¨urk¸ c¨u et al. [26], another proposal that will attract attention is the combination of the abovementioned model with the PID controller. In the model of Jia et al. [27], I can replace the PID and PI^2^ controllers with the adaptive controller or the optimal controller and I investigate their performance. Based on the results obtained after I calculated the parameters of the controller, I simulated them and I identified (SBL) according to the simulated graph, based on the model of the paper of Tan et al. [28]. From the results obtained by the simulated graphs, I can use them to design the controller (the inverse formula of the formula in the paper of Tan et al.). According to the paper of Sharma et al. [29], I will apply the controller to the system that I study about in the near future with communication time delay. Based on the paper of Sharma et al. [30], the new application can be considered for the adaptive controller. I can apply the optimal control method for the switching system as in [31]. The application of the fuzzy control method for the switched nonlinear system in [32] is a new idea. Applying the neuron control method to the invariant system [33] is a creative idea. I can apply the adaptive fuzzy control method for the nonlinear system in [34] to create something new for the research. I can use the optimal critic design method for the system in [35], and from there, I can find the advantages of the abovementioned method compared to the previous traditional methods. A new proposal in [36, 37] is to use this model in zero gravity, namely, it will work in space. The new approach in [38] is to use this model for drones.

In this paper, parameters of the survey are obtained by simulation software, so that the overall gimbal stabilization loop will achieve a relatively stable signal level through the evaluation of simulation results. I also use simulation results from 2 different methods to get a more accurate view of the system properties. Through this survey, I can clearly understand the stability of the system from the results achieved, which has not been previously indicated on a large scale. The advantages of the method presented below are easy-to-survey nature, high accuracy results for any complex system, and saving survey time compared to other methods. The disadvantage of this method is due to its easy-to-survey nature, so it cannot fully meet the requirements set out for each specific case. The rest of this paper is organized as follows. The modeling of the single-axis gimbal system is given in Section 2. Powerful adaptive controller design is presented in Section 3. Simulation results and discussion are presented in Section 4, and finally, conclusions are drawn out in Section 5.

## 2. Modeling of the Gimbal Actuator

### 2.1. Experimental Setup

**Figure 1:**
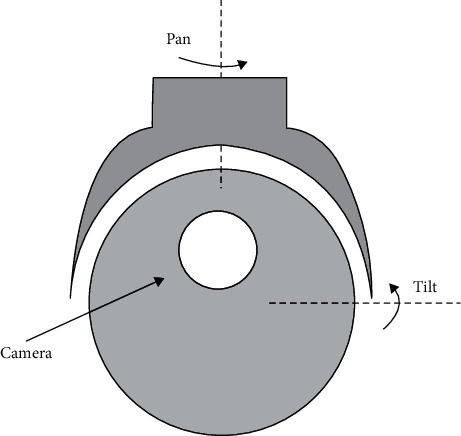
Front view of the yaw-pitch gimbal; a camera is mounted on the top of the inner gimbal.

**Figure 2:**
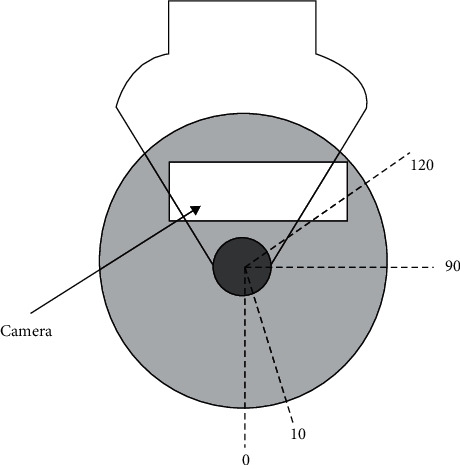
Pitch angle limit.

Figure 1 shows a structure that consists of a circular button that I can adjust in two directions: yaw (rotate) and pitch (tilt). The nature of the structure has some disadvantages; the camera is integrated with the gimbal inside, and the camera lens is oriented from the inside out. Therefore, the internal gimbal has an unbalanced mass due to the addition of the camera. Hence, adjusting the balance of the internal motor of the system is related to the pitch angle. Pitch angle is an important factor that needs to be investigated to be adjusted to suit usage needs. The gyroscope is mounted inside the gimbal with a two-axis ratio intended to measure the movement speed in two distinct directions, and it can be tuned to achieve uniform coordination between the camera and the system. The gyroscope signals communicate with a motor's torque, and the motor model is connected in series with the gimbal, forming the control system. The purpose of this control is to help stabilize the operation of the system and to create an interaction balance in a certain proportion between the components inside the system (regulator, monitor). The camera frame is masked by the external gimbal, and it is in front of the camera at pitch angles more than 120 degrees. Additionally, the images are inverted when they are displayed on the camera with an angle less than zero. Therefore, the pitch angle is limited by Figure 2. The design process must be accurate, and it always keeps the image subtle. To make it easy to take pictures and prevent blurring, the movement of the camera must be smooth and soft. In the field of automatic control, this must occur through a quick and precise response.

### 2.2. Gyroscope Model and Motor Model

The gyroscope sensor is used to measure the output angular rate or LOS rate of the gimbal. Typically, a gyroscope model is represented as a second-order transfer function given as

(1) $$\begin{array}{c} G_{g}\left(s\right)=\frac{\omega_{g}^{2}}{s^{2}+2\xi_{g}\omega_{g}s+\omega_{g}^{2}}, \end{array}$$

where *ω*_*g*_ and *ξ*_*g*_ are the natural frequency and damping ratio of the gyroscope, respectively.

The motor model transfer function is given by

(2) $$\begin{array}{c} G_{m}\left(s\right)=\frac{K_{m}}{s^{2}+2\xi_{m}\omega_{m}s+\omega_{m}^{2}}. \end{array}$$

### 2.3. Linearization of System Identification

**Figure 3:**
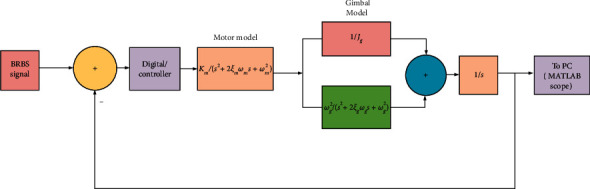
Block diagram of closed-loop identified linear mode.

A good practice skill for controlling a nonlinear system is to linearize the system using classical methods such as establishing equations of state, and I then select modern controllers for the system according to the individual theme. This section is devoted to the design of a controller for a common operation as outlined above, which can be divided into two parts: system identification through the actual model, and I set up the controller for the system. In the identity of the system, the system operating processes are represented by a block diagram with linear continuous time; then, the controller parameters are selected depending on the purpose of use. The model will determine the position of the controller in the block diagram, and the forward gain of the controller can be calculated directly experimentally to achieve the desired result; meanwhile, I can also adjust the controller parameters to preview the results through theoretical analysis. In this section, I have shown the comparison results when the system is not using any controller. The model of the system is shown in Figure 3.

## 3. Strong Adaptive Controller Design

The motor transfer function *G*_*m*_(*s*) and gyroscope transfer function *G*_*g*_(*s*) can again be written as

(3) $$\begin{array}{c} G_{m}\left(s\right)=\frac{0.85}{s^{2}+80\;s+2500},\\ G_{g}\left(s\right)=\frac{2500}{s^{2}+70\;s+2500}. \end{array}$$

*G* (*s*) of the system is given by

(4) $$\begin{array}{c} G\left(s\right)=\frac{73.92{\;s}^{2}+5174\;s+1.86\times 10^{5}}{s^{5}+150{\;s}^{4}+10600{\;s}^{3}+3.75\times 10^{5}s^{2}+6.2\times 10^{6}s+1.8\times 10^{5}}. \end{array}$$

## 4. Simulation Results and Discussion

**Figure 4:**
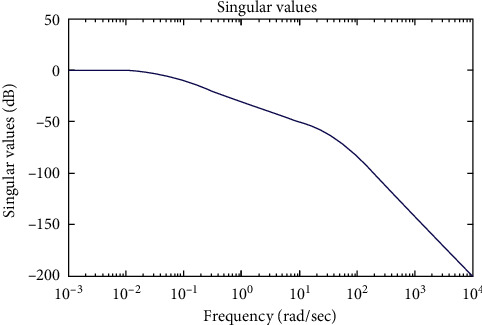
The Bode plot.

**Figure 5:**
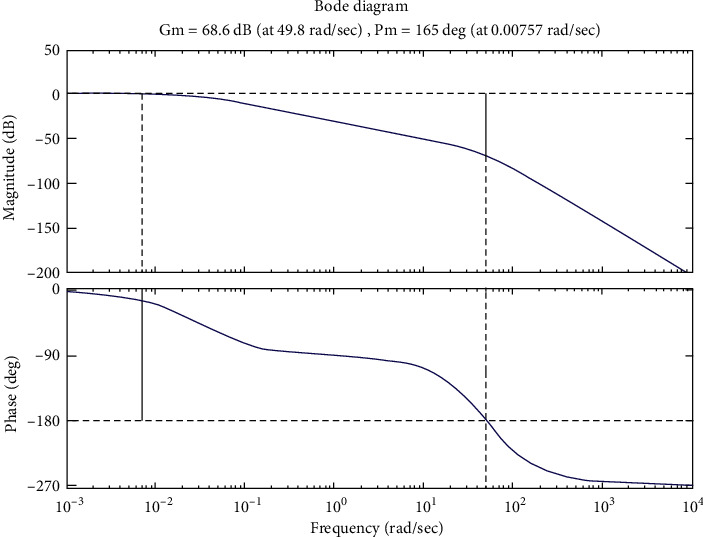
Bode plots represent marginal reserve values and phase reserve values.

**Figure 6:**
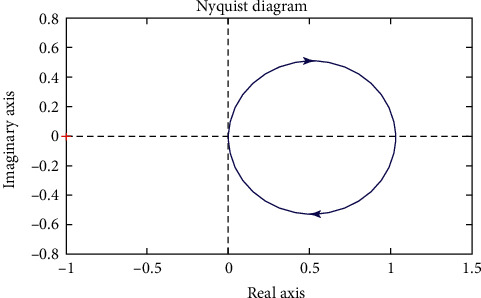
The Nyquist plot.

**Figure 7:**
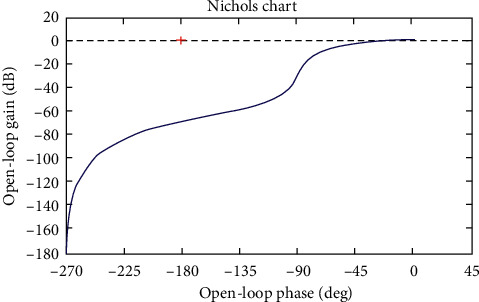
The Nichols plot.

Simulations of the system are shown in Figures 4–7.

Figure 4 shows the system degradation values. The profiles that ensure stability are given in the system as the minimum degradation value (−25dB) is large (10^−0.3^–10^0^ rad/s) at low frequencies (10^−3^ rad/s–10^0^ rad/s) and the maximum singularity value (−50 dB) is small (10^0^ rad/s) at high frequencies (10^0^ rad/s–10^4^ rad/s).

Stability conditions of the system based on the Bode plot are

(5) $$\begin{array}{c} \left\{\begin{array}{c} GM>0,\\ \varphi M>0. \end{array}\right. \end{array}$$

According to the Bode plot (Figure 5), I found

(6) $$\begin{array}{c} \left\{\begin{array}{c} GM=68.6\;dB\;>0,\\ \varphi M=165^{0}>0. \end{array}\right. \end{array}$$

So, the system is stable.

From 2 analytical methods (Figures 4 and 5), it shows that the system operates stably and there is no indication that the stability of the system is poor. Thus, modern control methods for the system, namely, the control algorithm, will be built based on the stability of the system.

My comments:

The results in Figure 5 have a more intuitive look and have more detailed data than in Figure 4.

Simulation results from the two abovementioned methods have correctly reflected the nature of the problem. (Table 1)

## 5. Conclusions

In this paper, a survey of the stability of a two-axis gimbal system for the camera based on the parameters of the Bode plot is proposed to confirm the assurance of system stability as well as helping the system achieve the desired function. Besides, the simulation helps me to determine the values of the parameters to have the basis of controlling the system's operation to suit the requirements. This also shows the flexibility in adjusting the parameters of modern controllers as it is applied to the abovementioned model. Through this survey, the achievements in researching modern control theory for stable systems such as the abovementioned system will be applied in the future. If control algorithms of nonlinear and unstable systems are a conundrum that cannot be solved in the near future, the control algorithms for the stable system will be a preferred resolution case. In the future, I can apply modern control algorithms such as adaptive control using neural networks in the abovementioned model.

## Figures and Tables

**Table 1:** Motor and Gyroscope specifications.

Natural frequency (*ω*_*n*_)	50 Hz
Motor model-damping ratio (*ξ*_*m*_)	0.8
Torque constant (*K*_*m*_)	0.85 Nm/A
Back EMF constant (*K*_*e*_)	0.85 V/rad/s
Total inertia (*J*)	2 kg·m^2^
Armature resistance (*R*)	4.5 Ω
Armature inductance(*L*)	0.003 H
Gimbal model-damping ratio (*ξ*_*g*_)	0.7

## Data Availability

Readers are free to access supporting data for research conclusions from the references at the end of this paper.
